# Editorial: Online prosocial behavior and altruism in adolescence and youth

**DOI:** 10.3389/fpsyg.2024.1402979

**Published:** 2024-05-03

**Authors:** Yolanda Pastor, Manuel Martí-Vilar, Michelle F. Wright, Lucas M. Rodriguez, Cesar Merino-Soto

**Affiliations:** ^1^Department of Psychology, Rey Juan Carlos University, Móstoles, Spain; ^2^Departamento de Psicología Básica, Universitat de València, Valencian, Spain; ^3^Department of Psychology, Indiana State University, Chicago, IL, United States; ^4^Instituto de Filosofía, Austral University, Buenos Aires, Argentina; ^5^Departamento de Psicología, University of San Martín de Porres, Chiclayo, Peru

**Keywords:** online prosocial behavior, altruism, adolescence, youth, psychosocial determinants

In recent decades, much of the research on adolescents and young people and their use of social networks has focused on abuse and risk, with less attention paid to positive actions like prosocial behavior (Erreygers et al., 2018; Lysenstøen et al., 2021). Social networks currently constitute a very important space for the socialization and development of adolescents and young people. In response, many scholars are beginning to emphasize the interdependence of online and offline life as young people develop from infancy to young adulthood (Lavertu et al., 2020; Nesi et al., 2020; Armstrong-Carter and Telzer, 2021). Some authors such as Nesi et al. (2020) have gone a step further, showing that the digital context is transforming how some milestones in adolescent development are produced, such as the construction of identity and body image and the quality of intimate relationships.

Social networks, then, create a situation in which prosocial behavior and altruism can be learned and developed from a very early age, fostering offline prosociality (Lavertu et al., 2020). Online prosocial behavior has been defined as “voluntary behavior carried out in an electronic context with the intention of benefitting particular others or promoting harmonious relations with others” [SIC] (Erreygers et al., 2018, p. 3). In social networks, prosocial behavior can take various forms, from giving a “like” to providing instrumental help, offering information of various kinds, showing support and comfort to others, offering help and support in the face of virtual aggression or threat or even promoting an altruistic cause or collecting funds for vulnerable groups.

This Research Topic calls attention to the need to study online prosocial behavior from childhood to young adulthood and to put this issue on the research agenda. At the same time, it emphasizes the interdependence between these behaviors and offline life.

The Research Topic presents four articles that demonstrate the importance of studying prosocial behavior in online contexts.

The first work aims to understand the variables that influence the prosocial behaviors of adolescent cyberbullying bystanders, for instance behaviors that protect the bullied (Cui and Li). The behavior of bystanders with regard to online violence is vital to curbing these attacks and protecting people from them. The results of this study show that family functioning is a key determinant of prosocial victim-supportive behaviors by adolescents. The higher the level of family functioning, the higher the level of empathy and social support shown by the adolescents, which in turn leads to an increased expression of supportive behaviors toward cyberbullying victims. Additionally, these relationships are more powerful in the case of girls. This research highlights the importance of family functioning in promoting prosocial behaviors in cyberbullying bystanders.

The second study, by Iwasa et al., explores the extent to which online and offline prosocial behaviors contribute to one of the main challenges faced by adolescents: the development of their own identity. Their results indicate that both online and offline prosocial behaviors contribute to identity development. Digital environments constitute spaces that can foster identity development by providing individuals with room to develop a sense of responsibility, roles and agency in society. As Iwasa et al. observe, “online prosocial behavior may encourage adolescents to consider and choose their future lives in society as identity options.” Teenagers' online interactions can be beneficial as they explore their identities, helping them to express themselves freely and examine their values without the social constraints of offline life. In line with co-construction theory, the study of adolescent development must take into account the interdependence of digital scenarios and offline life (Subrahmanyam et al., 2006, 2008).

The third contribution to this Research Topic examines how the framing of a social media campaign and individual differences can influence the intention of college students to make an online donation to an organization that supports children in vulnerable situations in third-world countries (Lee and Chu). The results confirm a greater intention to donate money when the advertisement for a campaign presents what the donation money will be spent on in fractions. Youth with a high need for cognition and those exhibiting a promotion-focused, self-regulatory approach expressed a greater intention to donate money when the advertisement partitioned the donation amounts for specific purposes. By contrast, youth who exhibited a prevention-based self-regulating approach were more likely to make a partitioned donation if they perceived the non-profit organization to be sincere and authentic.

The fourth paper explores the effect of brief mindfulness-based interventions—one face-to-face and one digital—on adolescents' online charitable behavior and the development of empathy, social connectedness, and self-compassion. The authors found that mindfulness interventions produced an improvement in empathy and compassion, which in turn led to increased online charitable behaviors in adolescents, with face-to-face interventions having a greater effect than digital interventions (Hong et al.).

These studies highlight the importance of analyzing prosocial behavior in online contexts and its interdependence with psychosocial development and offline life during adolescence and young adulthood. Development from infancy to young adulthood occurs in multiple interconnected settings, and the digital context is taking on greater prominence, transforming and feeding back into offline life. Further research on online prosocial behavior is needed to understand the role of an increasingly pervasive condition of human life: the online context.

## Author contributions

YP: Writing – original draft. MM-V: Writing – review & editing. MW: Writing – review & editing. LR: Writing – review & editing. CM-S: Writing – review & editing.
